# 'A Down and Out Eye': Case Report of a Primary Ethmoid Mucocele

**DOI:** 10.7759/cureus.14432

**Published:** 2021-04-12

**Authors:** Danielle James, Stephen Garry, Mel Corbett, Ivan Keogh

**Affiliations:** 1 Otolaryngology - Head and Neck Surgery, University Hospital Galway, Galway, IRL; 2 Otolaryngology, University Hospital Galway, Galway, IRL

**Keywords:** mucocele, endoscopic sinus surgery, ophthalmology, rhinology

## Abstract

Paranasal sinus mucoceles are benign, locally expansile masses of the paranasal sinuses that are lined by epithelial cells. They result from obstruction of sinus ostia. The close proximity of paranasal sinus mucoceles to the orbit and skull base predisposing the patient to significant morbidity. We describe the case of a previously healthy 23-year-old gentleman presenting with a five-day history of unilateral (left) eye pain and swelling with an obvious deformity. There was also no history of trauma or prior surgery. He underwent a CT sinus, which showed near complete opacification of the left anterior ethmoid sinus with bony destruction and obvious displacement of the orbit both laterally and anteriorly. This was assessed as to be in keeping with an ethmoid mucocele. Endoscopic marsupialization has become the preferred surgical approach over obliterative procedures for the treatment of paranasal sinus mucoceles. Primary ethmoid mucocele is an uncommon entity, especially in the absence of prior ear, nose and throat (ENT) complaints, and therefore should remain an important differential when a patient presents with a unilateral swelling causing proptosis.

## Introduction

Paranasal sinus mucoceles are epithelium-lined cystic masses usually resulting from obstruction of sinus ostia [1]. Mucoceles are benign, locally expansile masses of the paranasal sinuses. These benign, locally expansile masses lined by the mucoperiosteum of the sinus cavity may erode into the intraorbital or intracranial spaces which intuitively may go on to cause unwanted complications and morbidities [2]. These patients most commonly present following operative intervention to the sinuses such as a functional endoscopic sinus surgery (FESS) or as a complication of chronic rhinosinusitis. While ophthalmologic symptoms are the most frequent presentation, patients also report rhinological or neurological complaints. The close proximity of paranasal sinus mucoceles to the orbit and skull base predisposes the patient to significant morbidity [3]. Herein, we report an unusual case of a primary ethmoid mucocele with no prior ear, nose and throat (ENT) complaints.

## Case presentation

**Figure 1:**
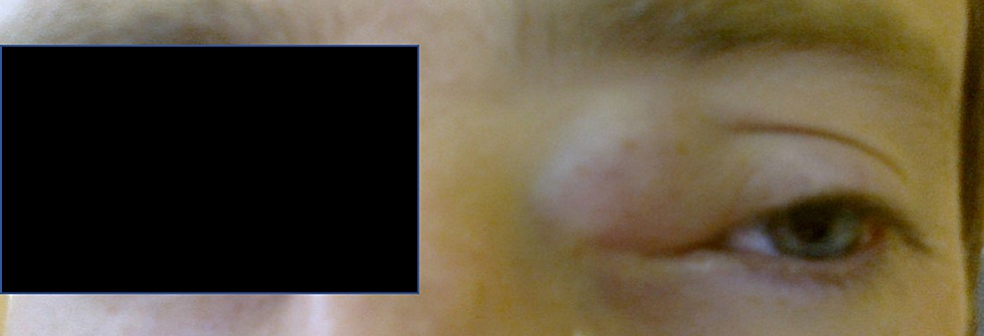
Clinical photograph showing swelling related to the left medial canthus.

A previously healthy 23-year-old gentleman presented to the Ophthalmology emergency room following referral by his General Practitioner (GP) with a five-day history of unilateral (left) eye pain and swelling with an obvious deformity. On further questioning, the patient had been complaining of rhinorrhoea and worsening blurred vision, but no symptoms of nasal congestion or hyposmia. He was systemically well and did not report nausea, vomiting, fevers or anorexia. There was also no history of trauma or prior surgery. Clinical examination revealed an obvious swelling related to his left medial canthus (Figure 1). This was soft, fluctuant, non-tender, and non-pulsatile. It was causing downward, lateral displacement of his eye. There was no surrounding erythema and no obvious punctum. There was no diplopia or nystagmus noted during his ophthalmological assessment and his visual acuity was normal.

**Figure 2:**
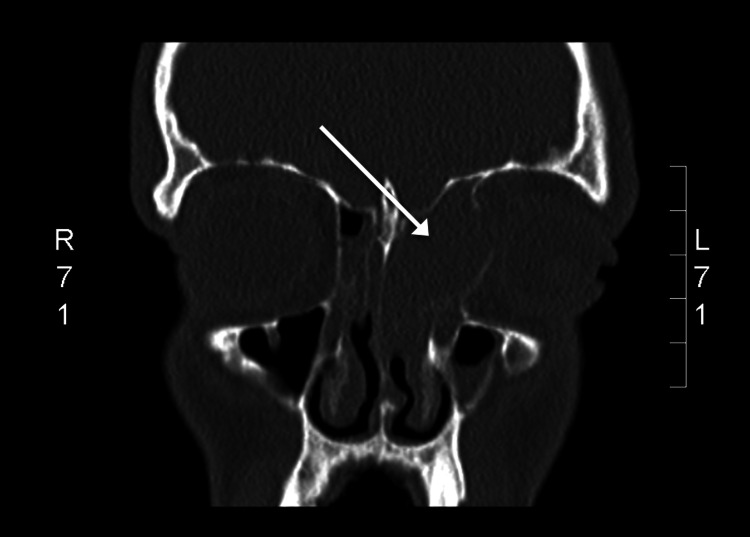
Coronal CT sinus showing near complete opacification of left anterior ethmoid sinus with obvious bony destruction. The mass effect on the left orbit is obvious with deviation to the left and the globe pushed anteriorly.

**Figure 3:**
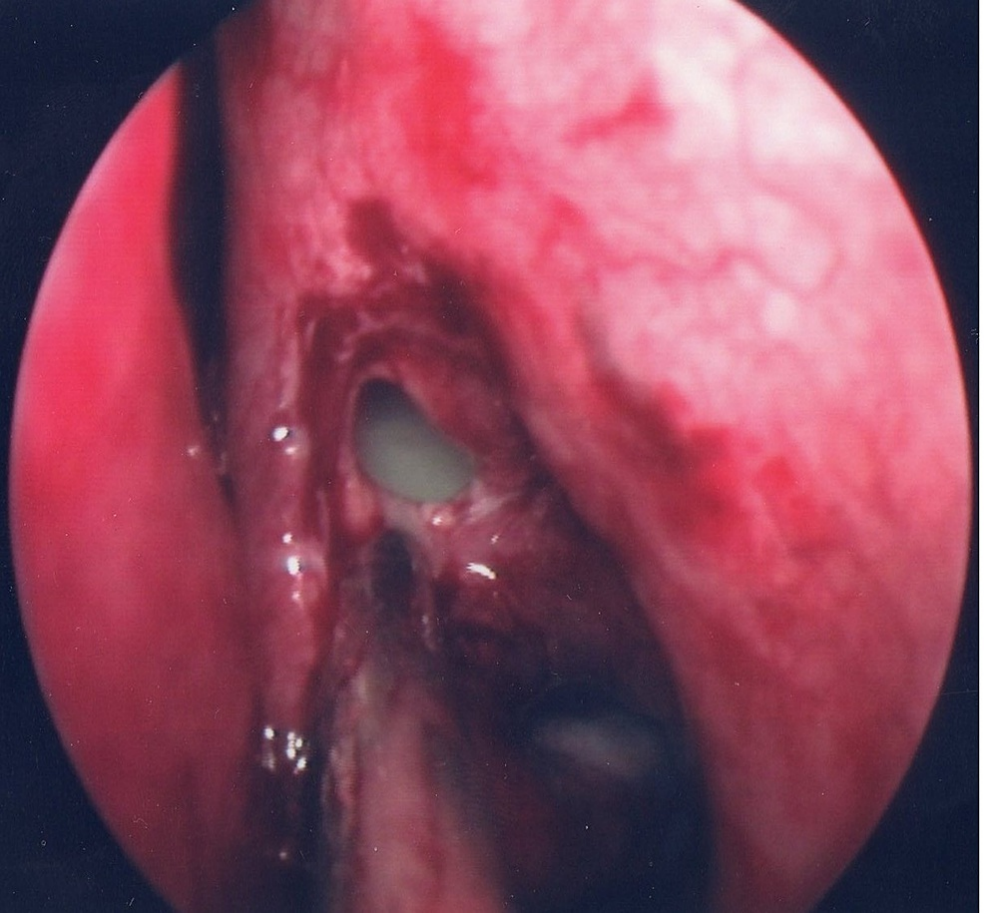
Post endoscopic decompression showing de-roofing of mucocele.

The patient was admitted and commenced on empirical antibiotic therapy, steroids, and topical nasal decongestants. He underwent a CT sinus, which showed near complete opacification of the left anterior ethmoid sinus with bony destruction and obvious displacement of the orbit both laterally and anteriorly (Figure 2). This was felt to be most in keeping with a primary ethmoid mucocele. Multidisciplinary input was sought (ENT, Radiology, Ophthalmology) and it was felt to be amenable to endoscopic drainage. The patient was brought to theatre and underwent an endoscopic septoplasty and turbinate reduction to improve access. He subsequently underwent surgical decompression, de-roofing, and marsupialization of the mucocele (Figure 3). Intra-operatively, there was obvious bony destruction to the medial wall of the orbit based on the tactile feedback given during instrumentation. The cyst was incised and thick, mucous-type fluid was drained. An aspirated sample was taken for microscopy, culture, and sensitivity as well as some tissue for histology. Postoperatively, he underwent a successful recovery with complete resolution of the proptosis and had a normal ophthalmological exam. He was discharged home four days post-operatively on antibiotics and a short course of topical decongestants. 

Microscopy of the aspirate showed coagulase-negative Staphylococcus. The histopathological report of the biopsy demonstrated benign respiratory epithelium with inflammation and mucinous glands within the submucosa which is consistent with a mucocele. He was recently reviewed in his three-month follow-up and has recovered fully, without residual functional deficits.

## Discussion

Mucoceles are the most common lesions causing expansion of the paranasal sinuses [4]. There exist multiple etiologies of mucoceles which include chronic infection, allergic sinonasal disease, trauma, and prior sinus surgery; however, in many cases, the cause remains unknown [5]. Mucoceles can be classified as either primary or secondary, with the latter frequently being related to prior FESS or sinus surgery. Secondary mucoceles following sinus surgery generally develop as a delayed complication, typically 10 to 30 years post-operatively [5]. There is a wide variation of symptoms that patients present with however facial pain, rhinorrhea, headache, and eye symptoms are among the most common. Regarding primary mucoceles, these are less commonly reported however they are most commonly found in the frontal and ethmoidal sinuses [1]. What is unusual about our case is that the patient had no previous ENT issues that being prior surgery or symptoms of chronic rhinosinusitis.

In our case, the presentation of a downwardly, laterally displaced eye is explained by the location of the mucocele. It is not uncommon for an ethmoid mucocele to present with an ophthalmological complaint. One large series reported that over 80% of patients with mucoceles presented with some degree of proptosis, and another found that 70% of patients presented initially to an ophthalmologist for evaluation [6].

In this respect, there was no question that the patient warranted surgical intervention. Endoscopic marsupialization has widely become the favoured surgical approach over obliterative procedures for the treatment of paranasal sinus mucoceles. This is due to reports of lower complication and recurrence rates as per much of the published data on the subject [6]. Open or combined approaches are often reserved for cases with extensive intraorbital extension [2].

## Conclusions

Primary ethmoid mucocele is an uncommon presentation especially with no prior ENT complaints and therefore should remain an important differential when a patient presents with a unilateral swelling causing proptosis. These cases are often managed endoscopically with endoscopic sinus surgery and therefore ENT surgeons should be familiar with the work up and management thereof.
